# Public Healthcare Procurement Strategies in Response to the COVID-19 Pandemic: A Scoping Review

**DOI:** 10.34172/ijhpm.8556

**Published:** 2025-08-10

**Authors:** Pierre-André Hudon, Matthew T. Haren, Jean-Baptiste Gartner, Frédéric Bergeron, André Côté

**Affiliations:** ^1^Département de Management, Faculté de Sciences de l’administration, Université Laval, Québec City, QC, Canada.; ^2^Centre de Recherche en Gestion des Services de Santé, Université Laval, Québec City, QC, Canada.; ^3^Centre de Recherche de l’Institut de Cardio-Pneumologie de Québec, Université Laval, Québec City, QC, Canada.; ^4^Centre de Recherche du CHU de Québec, Université Laval, Québec City, QC, Canada.; ^5^Centre de recherche du CIUSSS de Chaudière-Appalaches, Québec City, QC, Canada.; ^6^VITAM, Centre de Recherche en Santé Durable, Université Laval, Québec City, QC, Canada.; ^7^Bibliothèque-Direction des Servicesconseils, Université Laval, Québec City, QC, Canada.

**Keywords:** Public Procurement Strategies, COVID-19 Pandemic, Crisis Procurement, Healthcare Procurement, Public Procurement Environment, Healthcare Supplies

## Abstract

**Background:**

The COVID-19 pandemic posed unprecedented public healthcare procurement challenges. The objective of this review was to identify and characterise the scope of the literature on public procurement strategies for healthcare supplies during the COVID-19 pandemic (2019–2023) in relation to the public procurement contexts, systems, and processes and methods (the public procurement ecosystem) worldwide.

**Methods:**

We performed a scoping review of governmental strategies for the procurement of medical equipment, personal protective equipment (PPE), or medications related to the COVID-19 pandemic. Extracted data were mapped to the fields of the public procurement ecosystem. We used inductive thematic analysis to derive within-field themes, and subsequently, cross-cutting themes through which we structured a narrative synthesis.

**Results:**

1909 unique studies were identified through a systematic search, of which 89 met the inclusion criteria. One hundred and ten themes were derived from the extracted data within the 21 fields of the public procurement ecosystem, and from these, 10 cross-cutting themes were identified which served to structure the narrative synthesis. It was clear in this literature that the scale and impact of the COVID-19 pandemic required governments to act well outside of the public procurement processes and methods themselves, to procure and distribute the required supplies. Notwithstanding the significant attention to contextual and system-level responses, there were significant responses at the procurement process and methods level, including rapid and temporary expedited procurement processes and longer-term strategic procurement responses.

**Conclusion:**

This scoping review of public procurement strategies during the COVID-19 pandemic has demonstrated a focus of the literature not only on the public procurement processes and methods themselves, but also on governmental actions to adapt both structures of public procurement systems and conditions within broader environmental contexts to facilitate procurement goals.

## Background

 The COVID-19 pandemic is generally considered to have spanned from late 2019 through early 2023. The first known cases were reported in Wuhan, China in December 2019. World Health Organization (WHO) declarations of COVID-19 as a Public Health Emergency of International Concern (January 30, 2020) and as a pandemic (March 11, 2020) followed. The WHO officially declared an end to COVID-19 as a global health emergency on May 5, 2023, marking a major milestone in the pandemic response.^1^ This timeline reflects the period during which COVID-19 was treated as a global public health crisis, although the virus continues to circulate and pose health risks.^2^

 Early in this period, public health and emergency systems around the world had to shift rapidly and radically to address the public health threat of the novel coronavirus.^3^ Due to the global scale of response actions such as mass screening and diagnostic testing,^4,5^ the ubiquitous public use of face masks^6^ and hand sanitization, and the needs of hospitals running at capacity, demand-side pressures on personal protective equipment (PPE) and healthcare supplies, challenged complex global healthcare supply chains.^7^ Thus, public procurement strategies for healthcare supplies emerged at the forefront of governmental responses to the COVID-19 pandemic.

**Figure 1:**
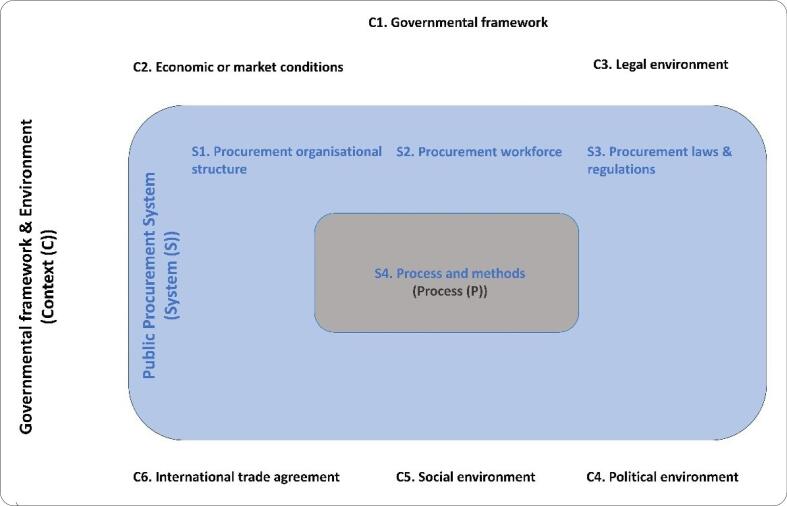
Procurement Processes and Methods Within a Public Procurement System Operating in a Governmental Framework and Environment.

 Public procurement processes take place within a public procurement system, which operates within governmental frameworks and environments with cultural, administrative, economic, legal, and social domains.^8^ Thus, to explore the procurement-related actions of governments during the COVID-19 pandemic, we must first define these three levels (Figure 1).

 Firstly, the procurement process begins with determining specifications and moves through selecting suppliers, contracting, ordering, expending and evaluation, and ends with follow-up and evaluation.^9,10^ These processes and methods form one of the four pillars which define the public procurement system. The other three pillars are procurement laws and regulations, procurement workforce, and procurement organisational structure.^8^ Lastly, the governmental framework and environment dictate the public procurement system. For example, in a unitary governmental system, local government procurement structures and processes are dictated by the national government, whereas in federal government systems, state and local governments have a high level of autonomy to create their own procurement structure, methods and processes.^8^ In both government systems, public procurement organisations at each level of government can be centralised or decentralised. Government frameworks operate within cultural, administrative, economic, legal, and social environments. Within these, economic or market conditions have an influence on the effort of public procurement systems to maximise competition. The legal environment refers to the broad legal framework that governs all business activities. The political environment refers to the interests, objectives, and beliefs of groups, policy-makers and management and their influence over procurement statutes, budget authorisation, and appropriation processes. The social environment constitutes media, civil society, local community engagement, and the independence of the citizenry, which together holds procurement officials accountable for transparency, fairness and efficiency.^8^ The last field of the context within which public procurement systems operate is international trade agreement and World Trade Organization codes, which cover reshipment inspections, rules of origin, and technical barriers to trade.^8^

 The management of public procurement strategies, according to the framework of Schapper and colleagues, involves trade-offs between policy objectives or outcomes, conformance to regulations and fair dealing, and efficiency and value for money.^11^ In crisis or emergency situations, the risk of negative effects from trade-offs can be managed through preventive safeguards established before the emergency occurs, such as framework agreements.^12^

 By better understanding the range of public procurement strategies and actions adopted during the COVID-19 pandemic, opportunities for the development of new safeguards, improved management or reformation of public procurement of healthcare supplies in pandemics and similar crises may be identified. Therefore, this research seeks to understand the concepts that underpinned public procurement strategies for healthcare supplies during the management of the COVID-19 pandemic across levels of government. This research is interested not only in the procurement process itself, but also in any strategies or actions taken by governments to influence the procurement system or the broader procurement environment to facilitate the procurement process for healthcare supplies during the COVID-19 pandemic.

 The opportunity for knowledge development in relation to governmental responses to procurement challenges lends itself to a scoping review methodology for the following reasons: (1) the literature is new and has emerged rapidly; (2) the phenomenon is global and shared, but the governmental responses were necessarily rapid and concurrent, allowing little opportunity to calibrate and benchmark across jurisdictions; which provides us with; (3) an opportunity to map the key concepts in the literature that underpinned governmental responses worldwide, to clarify working definitions and conceptual boundaries, and illustrate both what has been learned and can be implemented in practice, and the gaps in knowledge that should drive future research.^13^

 A preliminary search of the Cochrane Database of Systematic Reviews, JBI Evidence Synthesis, and PROSPERO identified one published scoping review protocol^14^ but no completed reviews. We followed the Preferred Reporting Items for Systematic reviews and Meta-Analyses extension for Scoping Reviews (PRISMA-ScR) guide^15^ and adopted the recommended Population, Concept, Context mnemonic to develop the research question and objective statements^16^:

 “What were the procurement strategies (concept) for healthcare supplies (concept) that were used by governments (population) during the COVID-19 pandemic (context)?”

 Thus, the objective of this review was to identify and characterise the scope of the literature on procurement strategies for healthcare supplies that were used by governments and public administrations during the COVID-19 pandemic (2019–2023), to map the strategies, and identify the key learning and knowledge gaps to guide future research and practice.

## Methods

 To achieve this research objective, we performed a scoping review of the scientific literature on public healthcare procurement during the COVID-19 pandemic. The protocol has not been registered nor previously published.

###  Eligibility Criteria

 The eligibility, inclusion and exclusion criteria are shown in Table 1.

**Table 1:** Eligibility, Inclusion, and Exclusion Criteria by Population, Concept, Context

	**Eligibility**	**Inclusion**	**Exclusion**
(P) Population	Governments or public authorities, all levels, worldwide	Related to any level of government or public authority worldwide	Related primarily to private sector actions
(C) Concept	Procurement strategies or actions	Focussed on procurement strategies or actions	Insufficient in their focus on government procurement strategies or actions
	Medical equipment, PPE, COVID-19 medications	Related to procurement of medical equipment, PPE, COVID-19 medications	Not related to procurement of medical equipment, PPE or COVID-19 related medications
			Concerned only with the procurement of COVID-19 vaccines
(C) Context	Related to the COVID-19 pandemic	Reporting actions in response to the COVID-19 pandemic	Not related to the COVID-19 pandemic (H1N1, Ebola, etc)
Limits	Primary research	Primary research	Not primary research (eg, theses, editorials, commentary, opinions, review articles, etc)
			Written in languages other than English, French, Spanish, or Portuguese

Abbreviation: PPE, personal protective equipment.

###  Search Strategy

 A systematic search was developed in accordance with the PRISMA guidelines^15^ by an academic librarian (F.B.), to identify peer-reviewed studies published between 2019 (the beginning of the pandemic) and March 12, 2025 (the date of the last search). This timeframe ensures full and up-to-date coverage of the pandemic period. The search was designed around variations and combinations of the following terms: governments, public, state, hospitals (Populations); procurement, purchasing, supply chain, logistics, medical supplies, PPE (Concepts); COVID-19, coronavirus, pandemic (Contexts) as keywords and subject headings across the following databases: Medline (Ovid), Embase (Embase.com), Web of Science, ABI/Inform (ProQuest), and MedRxiv (medrxiv.org). The full search strategy along with Peer Review of Electronic Search Strategies (PRESS)^17^ and PRISMA-ScR^15^ checklists are provided in Supplementary file 1.

###  Selection of Articles

 We used Covidence^18^ to manage the record screening and review process, through which articles were first screened against the inclusion and exclusion criteria (Table 1) based on titles and abstracts, followed by full text screening of potentially relevant articles for the final inclusion decision. All screenings were performed by two independent reviewers. Disagreements were managed by discussion, moderated by a third independent reviewer, who made the final decision if consensus was not reached.

###  Quality Assessment

 Scoping reviews seek to develop a comprehensive overview of the evidence, rather than a quantitative or qualitative synthesis of data, and are not designed to underpin practice decisions. It is therefore not usually necessary to undertake a critical appraisal of sources.^19,20^ As such, we did not formally assess the quality of each selected article.

###  Data Extraction and Charting

 Extraction of data from full texts was performed by two independent reviewers according to our extraction chart (Supplementary file 2) based on predetermined categories: geographic location, level of government, empirical topic of research, research questions/objectives, hypotheses, methods, findings, conclusions and limitations, and public procurement strategies identified as very short-term, short-term, medium-to-long-term, and long-term strategies.

 Extracted government strategies were subsequently charted in the public procurement ecosystem defined in the introduction, incorporating the Governmental framework and environment, the public procurement system, and the public procurement processes and methods (Supplementary file 2).

###  Analysis

 Study context data (geography, level of government, and empirical research topics) were reduced to meaningful categories and analysed both descriptively and by multidimensional scaling to visualize relationships, using MS Excel and Orange v3.38.1.

 Using the data charted to the public procurement ecosystem (Supplementary file 2), we used inductive thematic coding to derive themes within each field and to map them to the public procurement ecosystem. We then explored the data and, again inductively, derived cross-cutting themes through which we structured and produced a narrative synthesis. This included, where applicable, commentary on the trade-offs within the procurement management framework of Schapper and colleagues,^11^ described previously.

## Results

###  Description of Selected Literature

**Figure 2:**
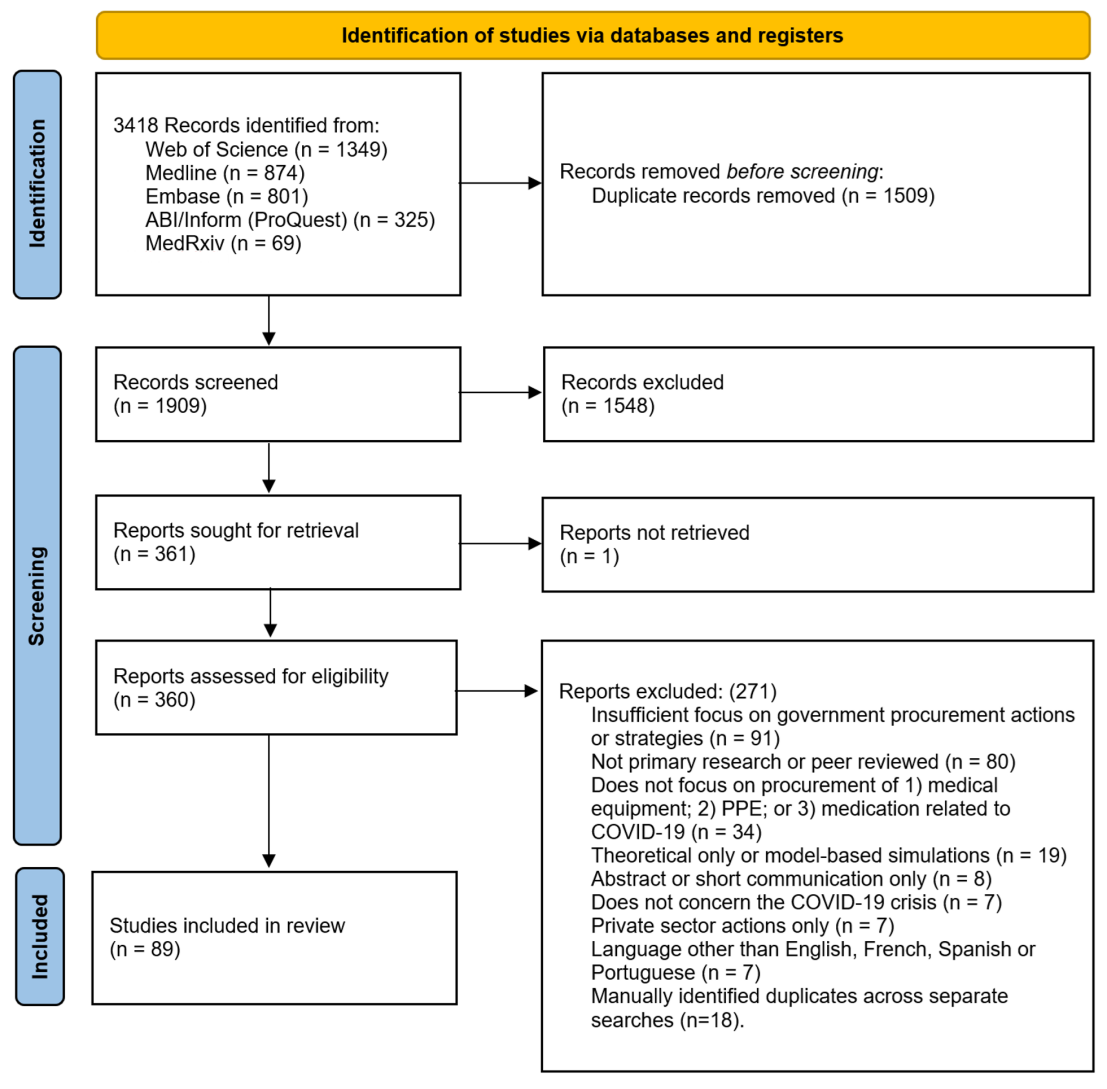
PRISMA Flowchart^21^ of Systematic Article Identification and Selection. Abbreviations: PRISMA, Preferred Reporting Items for Systematic reviews and Meta-Analyses; PPE, personal protective equipment.

Figure 2 shows the PRISMA flowchart. Of the 1909 records screened, 360 articles underwent full text assessment and 89 were included in this review ^[1]^.

**Figure 3:**
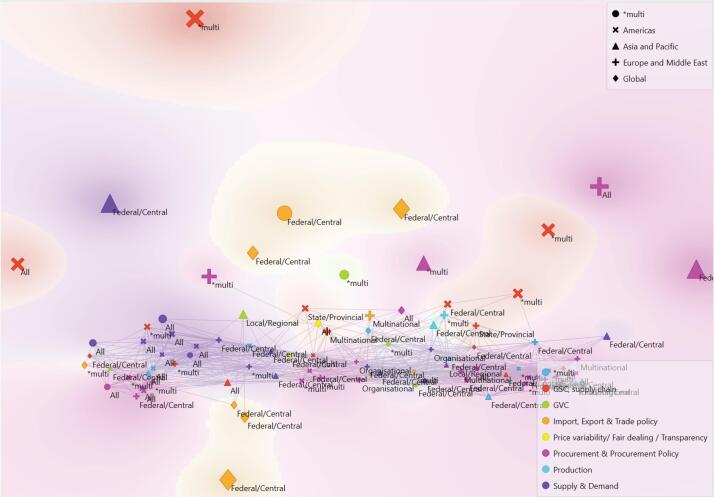
A Relational Map of the Selected Literature in Terms of Geography (Marker Symbols), Empirical Topic (Marker Colours) and Level of Government (Marker Labels). The more tightly clustered a group of papers is the more closely they are related to each other across these three dimensions. The gradients in area colour indicate the strength of and relationships between the empirical topics addressed in the selected literature. Abbreviations: GSC, global supply chain; GVC, global value chain.

 Eighteen studies were published in 2020,^7,22-38^ 35 in 2021,^39-73^ 23 in 2022,^74-96^ 5 in 2023,^97-101^ and 8 in 2024,^102-109^ twenty-seven studies addressed responses in the Americas,^7,22,25-27,32,39,42,45,46,48,49,55-58,72,74,75,88,92,97,102-105,107^ 24 across Europe and the Middle East,^30,35,41,43,50,51,59,60,62,66,68,69,73,78,79,87,90,93,96,98-101,106^ 16 across the Asia Pacific region,^33,36,37,44,53,54,65,67,71,81,83,86,89,94,95,109^ whilst 22 took a global (n = 14)^24,29,34,38,40,52,63,64,76,80,82,84,85,91^ or multi-country (n = 8)^23,28,31,47,61,70,77,108^ focus. Forty-three studies examined strategic responses at all (n = 20)^7,23,26,27,31-33,39,44,45,47-49,63,70,76,93,94,100,107^ or multiple (n = 23)^29,30,35,42,43,46,51,55,58,61,72-74,82,83,85,87,92,97,98,102,103,105^ levels of government. Thirty-four studies solely addressed the federal or central government responses,^22,24,25,34,37,38,40,41,50,52-54,56,57^, ^64,65,67,68,71,77,79-81,84,86,88,91,94-96,104,106,108,109^ whilst fewer papers specifically addressed multinational (n = 4),^59,62,90,99^ state/provincial (n = 2),^69,75^ local/regional (n = 3)^36,89,101^ and organisational (n = 3)^60,66,78^ responses. The primary research topics across the studies were procurement and procurement policy (n = 25)^23,26,27,35,41-43,55,57,60,63,68,70,73,78,83,90,93,95,98,99,103,104,106,107^; supply and demand challenges (n = 20)^28,31,32,37,39,45,47,48,50,51,53,54,65,66,79,81,89,97,105,109^; supply chain (including global supply chain) (n = 16)^24,30,33,46,49,58,59,69,72,74,75,88,91,92,94,100^; import, export & trade policy (n = 9)^29,34,38,40,52,62,77,84,85^; global value chains (GVCs)(n = 5)^25,36,61,64,82^; production (n = 2)^86,87^; price variability, fair dealing and transparency (n = 2),^56,76^ whilst 10 studies addressed multiple of these topics.^7,22,44,67,71,80,96,101,102,108^ A relational map of the selected literature in terms of geography, research topic, and level of government is shown in Figure 3. The dominant topics of procurement/procurement policy and supply and demand represented closely related literature and covered all levels of government and geography. In contrast, the import, export, and trade policy literature were more isolated, globally focussed and concentrated at the federal/central level of government. The supply chain (including global supply chain) literature, from across all geographies and considering multiple levels of government, appeared divided between articles related to the dominant topics and articles that were more isolated.

###  Governmental Procurement Strategies and Actions 

**Figure 4:**
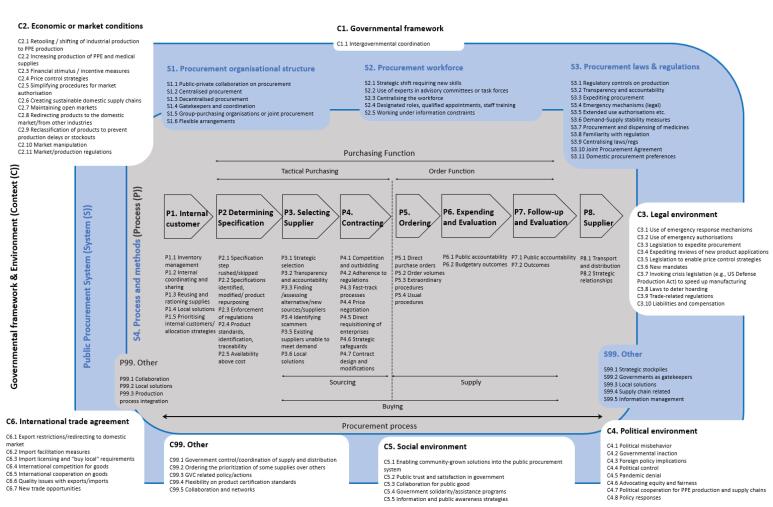
Strategy Areas Derived From the Selected Literature on Governmental Procurement Actions and Strategies During the COVID-19 Pandemic (2019 to 2023) Mapped to the Public Procurement Ecosystem. Figure adapted from Kumar et al,^9^ van Weele,^10^ and Thai.^8^ Abbreviations: PPE, personal protective equipment; GVC, global value chain.

Figure 4 summarises the extracted data from the selected literature as 110 strategy areas within the public procurement ecosystem.

 These strategy areas organised into 10 cross-cutting themes. Table 2 shows how these 10 themes cut across the 110 strategy areas within the public procurement ecosystem, which we now outline and, where possible, examine procurement management trade-offs.^11^

**Table 2:** Cross-cutting Themes Relating to Strategy Areas From the Selected Literature on Governmental Procurement Actions and Strategies During the COVID-19 Pandemic (2019 to 2023), Mapped to the Public Procurement Ecosystem

**Cross-cutting Theme**	**Ecosystem Reference**
Collaboration, cooperation, and coordination	C1.1 Intergovernmental coordination C4.1 Political misbehaviour C4.2 Governmental inaction C4.3 Foreign policy implications C4.4 Political control C4.7 Political cooperation for PPE production and supply chains C5.3 Collaboration for public good C6.4 International competition for goods C6.5 International cooperation on goods C99.1 Government control/coordination of supply and distribution S1.1 Public-private collaboration on procurement S1.2 Centralised procurement S1.3 Decentralised procurement S1.4 Gatekeepers and coordination S1.5 Group-purchasing organisations or joint procurement S2.2 Use of experts in advisory committees or task forces S3.10 Joint Procurement Agreement S99.2 Governments as gatekeepers P1.2 Internal coordinating and sharing P4.1 Competition and outbidding P99.1 Collaboration
Facilitating domestic PPE supply chains	C2.1 Retooling/shifting of industrial production to PPE production C2.2 Increased production of PPE and medical supplies C2.3 Financial stimulus/incentive measures C2.5 Simplifying procedures for market authorisation C2.6 Creating sustainable domestic supply chains C2.8 Redirecting products to the domestic market/from other industries C2.9 Reclassification of products to prevent production delays or stockouts C2.11 Market/production regulations C3.4 Expediting reviews of new product applications C3.7 Invoking crisis legislation to speed up manufacturing C99.2 Ordering the prioritisation of some supplies over others S3.11 Domestic procurement preferences P99.3 Production process integration
Market control strategies	C2.4 Price control strategies C2.7 Maintaining open markets C2.10 Market manipulation C2.11 Market/production regulations C3.5 Legislation to enable price control strategies S3.1 Regulatory controls on production S3.6 Demand-supply stability measures
Crisis legislation and emergency measures	C3.1 Use of emergency response mechanisms C3.2 Use of emergency authorisations C3.7 Invoking crisis legislation to speed up manufacturing S3.4 Emergency mechanisms (legal) S3.5 Extended use authorisations, etc S99.1 Strategic stockpiles
Expedited procurement	C3.3 Legislation to expedite procurement S3.3 Expediting procurement P2.1 Specification step rushed/skipped P2.2 Specifications identified, modified/product repurposing P2.5 Availability above cost P3.3 Finding/assessing alternative/new sources/suppliers P3.4 Identifying scammers P4.3 Fast-track processes P4.7 Contract design and modifications P5.1 Direct purchase orders P5.3 Extraordinary procedures
New strategic procurement	S2.1 Strategic shift requiring new skills S2.3 Centralising the workforce S2.4 Designated roles, qualified appointments, staff training S2.5 Working under information constraints P2.4 Product standards, identification, traceability P3.1 Strategic selection P3.3 Finding/assessing alternative/new sources/suppliers P4.6 Strategic safeguards P4.7 Contract design and modifications P5.2 Order volumes P5.3 Extraordinary procedures P5.4 Usual procedures P8.2 Strategic relationships
Trade measures	C3.9 Trade-related regulations C6.1 Export restrictions/redirecting to the domestic market C6.2 Import facilitation measures C6.3 Import licencing and "buy local" requirements C6.6 Quality issues with exports/imports C6.7 New trade opportunities C99.3 GVC related policy/actions
Equity, fair dealing, public trust	C3.1 Liabilities and compensation C4.6 Advocating equity and fairness C5.2 Public trust and satisfaction in government S3.2 Transparency and accountability P2.3 Enforcement of regulations P3.2 Transparency and accountability P4.1 Competition and outbidding P4.2 Adherence to regulations P4.4 Price negotiation P4.5 Direct requisitioning of enterprises P5.2 Order volumes P6.1 Public accountability P6.2 Budgetary outcomes P7.1 Public accountability P7.2 Outcomes
Local solutions	S99.3 Local solutions P1.4 Local solutions P3.6 Local solutions P8.2 Strategic relationships P99.2 Local solutions
Information and inventory management	S99.5 Information management P1.1 Inventory management P1.5 Prioritising internal customers

Abbreviations: PPE, personal protective equipment; GVC, global value chain.

###  Crisis Legislation and Emergency Measures

 Governments invoked crisis legislation and emergency measures to expedite procurement processes, mobilise production, and manage strategic stockpiles.

####  Invoking States of Emergency and Expedited Procurement

 The declaration of states of emergency by national and state/provincial governments activated emergency management legislation,^35,58,72,75,77,83,87,99,106^ which enabled expedited procurement processes and simplified regulations^35,55,66,75,78,83,99,106^ to meet urgent demands for PPE and medical supplies. For instance, in Ecuador, emergency contracting procedures required a formal declaration of a sanitary emergency, enabling state institutions to bypass traditional procurement processes.^55^ However, such emergency provisions were not always used to effectively expedite procurement. In the United States, despite an emergency declaration by the Secretary of Health and Human Services,^72^ the federal government did not issue a national emergency supply procurement measure, leaving states to compete for supplies on their own.^28^

####  Mobilising National Production Through Crisis Legislation

 Governments used crisis legislation to mobilise domestic production and address supply shortages. Nationalisation of production was implemented in various countries.^77,82,86,88,91^ The US invoked the Defense Production Act (DPA) multiple times, empowering the president to compel and fund manufacturers to produce critical supplies.^77,82,86,91^

####  Strategic Stockpiles and Replenishment Strategies

 While the DPA was effective in ramping up production, delays in its invocation contributed to the rapid depletion of the Strategic National Stockpile^7^ which was vulnerable due to inadequate maintenance.^7,72^ Efforts to replenish it were initiated through crisis legislation, including new legislation which sought to ensure that surplus stock produced under the DPA was used to restock it.^22^ Korea implemented similar strategies.^37^ In the US, the Strategic National Stockpile provided respirators, masks, and ventilators to New York during their state of emergency declaration.^58^

####  Emergency Authorisations and Regulatory Flexibility

 Emergency use authorisations (EUAs) and regulatory flexibility played crucial roles in ensuring the timely availability of medical supplies. The US Food and Drug Administration (FDA) extensively used EUAs to approve otherwise-unlicensed PPE, diagnostic tests, and therapeutics.^72^ They facilitated the repurposing of non-medical supplies and supported innovative solutions, such as the extended use and reprocessing of single-use PPE.^48,77^ Korea’s use of EUAs allowed small and medium enterprises to rapidly produce test kits, boosting domestic supply and export capacity.^67^ Similarly, the European Commission authorised the use of PPE that met alternative standards, expediting market entry.^60^

###  Expedited Procurement

 The range of existing and new legislation invoked to enable rapid procurement of critical medical supplies and PPE across countries led to varied adaptations of procurement processes to meet urgent needs.

 In the European Union (EU), countries invoked existing frameworks, such as the 2014 EU Directives, which allowed for negotiated procurement procedures without prior publication in situations of extreme urgency.^35^ This facilitated the rapid acquisition of essential materials like PPE and medical devices, often with flexibility in evaluation criteria (50% price, 50% quality, with delivery time as a key factor).^35^ An analysis of contract award notices published in the Tenders Electronic Daily during the first European wave of COVID-19 (January 2020 to September 2022) showed that awarding contracts by negotiated procedure without prior publication or call for competition was primarily used in March and April, and that from May to September there was a shift towards transparent open procedures. There was also relatively frequent use of framework agreements and the adoption of cooperative purchasing in countries such as Italy, Latvia, Estonia, and Norway.^99^

 Some countries, like Portugal, responded by introducing specific legislative measures tailored to the pandemic’s demands. This included an exceptional public procurement regime (March 2020), enabling authorities to make urgent purchases without adhering to standard procedures.^73^ Similarly, in Italy, the government enacted emergency measures allowing deviations from traditional procurement principles.^78^

 In the US, where states lacked federal support, those who centralised procurement, were able to negotiate directly with Asian suppliers and used corporate liaisons to help vet, negotiate, and arrange shipments with PPE manufacturers.^105^ The case was similar in the Netherlands, when wholesalers failed early in the pandemic, hospital buyers engaged in direct sourcing from manufacturers in East Asia.^100^ However, inexperience and rapid procurement timelines sometimes led to substandard product selections in such circumstances, including that of counterfeit PPE.^41,49,80,100^

 Findings from interviews with forty contract managers in the US, involved in purchasing PPE in the months following the president’s emergency declaration, revealed that contractor selection was not based on competitive factors but on vetting, with existing relations and track record being top of a narrow set of selection criteria.^103^ Consistent with this, only 4% of contracts for PPE in the first year of the pandemic in the US, were issued to first-time contractors, yet inconsistently, more than half of contracts were apparently awarded competitively.^104^ Almost all were developed as fixed-price contracts and 20% required some form of contract change (termination, unilateral modification, bilateral modification) during the contract period. Surprisingly, competitive contractor selection increased the likelihood of contract termination in this context.^104^

###  Trade Measures

 Countries employed a variety of measures to address critical shortages of medical supplies. Among the most prominent were export restrictions, which became widespread in early 2020. Within the EU, for instance, member states including France and Germany, imposed national bans on PPE exports to other EU countries, prompting the European Commission to step in and issue collective export authorisations.^34,38,40,62,77^ Similar actions were taken by other nations,^40^ including the US, which imposed restrictions on the export of respirators, masks, and other essential medical supplies and redirected them to the domestic market under the DPA.^7,77^ These measures were characterised by both short-term restrictions and longer-term policy shifts, with some countries, such as China, initially diverting domestically produced PPE to meet local demand before gradually resuming exports by June 2020.^77^ These measures reflected a broader trend of governments prioritising the containment of domestic shortages, with the Global Trade Alert reporting over 50 countries implementing such restrictions by March 2020.^34^

 As export controls proliferated, import facilitation measures emerged as another critical trade policy tool. To mitigate the effects of supply shortages, many countries took steps to lower tariffs and streamline customs procedures for the importation of medical supplies. The US, for example, reduced tariffs on medical products under the Section 301 tariff agreement with China,^77^ and several South American countries, such as Brazil, Colombia, and Paraguay, similarly lowered tariffs on PPE,^29,62^ as did European countries such as Greece.^106^ By the end of March 2020, more than 70 countries had introduced measures aimed at facilitating imports, such as removing import duties and expediting clearance processes.^84,85^

###  Market Control Strategies

 Various strategies were implemented across the globe to control market dynamics for essential medical supplies, including price controls, regulation of production and distribution, and adjustments to existing market structures.

####  Price Control Strategies

 Governments adopted price control measures to curb inflationary pressures on essential products like hand sanitizers, masks, and PPE. For instance, New York State leveraged prisoner labor to produce hand sanitizers, circumventing price hikes caused by supply chain disruptions.^58^ China and India imposed price caps and regulated wholesale prices of these goods under emergency provisions,^37,83,94^ as did European countries.^93^

####  Market and Production Regulations

 In response to critical shortages, several countries relaxed regulatory requirements to expedite the production of medical supplies. Canada, the US, the UK, and others, waived or fast-tracked approval processes for companies converting operations to produce medical goods, including ventilators and PPE.^23,47,58,86,91^ Temporary certifications, such as the CE mark for medical devices, were issued to accelerate the process of getting essential products to market.^58,86,91^

####  Market Manipulation and Distribution Control

 Despite these regulatory efforts, market manipulation and hoarding posed significant challenges. Stocks of medical supplies were frequently sold to the highest bidder, exacerbating the difficulties faced by governments and consumers.^76^ In response, several governments implemented rationing measures to stabilize supply and ensure equitable distribution. For instance, China set maximum quotas for individual mask purchases,^33^ and South Korea mandated that 80% of mask production be distributed through public channels.^37^

###  Collaboration, Cooperation, and Coordination

 The pandemic exposed significant challenges in government procurement processes and highlighted the need for enhanced collaboration, coordination, and strategic approaches which the literature showed were managed with varying degrees of success.

####  Intergovernmental Coordination, and Fragmentation

 During the pandemic, the lack of cohesive intergovernmental coordination, particularly in countries like the US, exacerbated procurement challenges. The federal government’s failure to leverage its procurement capacity led to intense competition between state, local, and federal authorities for essential goods like PPE.^57,58^ In contrast, countries such as Canada, Germany, and Switzerland demonstrated successful vertical and horizontal coordination, sharing resources and facilitating cross-level collaboration to optimize distribution.^70^

####  Centralised vs. Decentralised Procurement Approaches

 Governments adopted both centralised and decentralised procurement strategies with varying levels of success. Centralised procurement, as seen in Korea, Germany, Spain, and the EU’s Joint Procurement Agreement, amongst others, allowed for bulk purchasing, price stabilization, and equitable distribution.^35,37,59,66,92,93,96^ In Québec, during the early response phase a new simplified public procurement system emerged where the role of the Treasury Board was minimised and the Ministry of Health’s logistics directorate officially became the sole interface between the healthcare institutions and suppliers.^102^ Conversely, decentralised approaches in the US led to state-level competition and inflated prices.^7,23,26,27^ In Brazil, the success of joint public procurement related to the pandemic by four established horizontal interstate consortia was variable. The Consórcio Brasil Central, was the only one that achieved financial benefits from joint public procurement of medicines and PPE.^107^ This was attributed to the maturity of the consortium’s administrative and normative structure and processes relative to the newer consortia.^107^

 At the local or municipal level, a study of South Moravian region (Czech Republic) documented criticism from smaller authorities that the larger municipalities with extended authority under which they fall could have organized joint, centralised purchases of required supplies (such as disinfectants).^101^ In response, there were examples of ad-hoc joint-purchasing of larger volumes of products by neighbouring towns and municipalities, that were then redistributed in smaller lots.^101^

####  Public-Private Partnerships and Collaboration

 Public-private partnerships (PPPs) emerged as a crucial mechanism for addressing supply chain disruptions. In the US, innovative PPPs enabled local businesses and government entities to secure and distribute PPE effectively.^27,42^ Countries like South Korea and China successfully leveraged partnerships with private enterprises to expand testing capacities and ensure a stable supply of medical equipment.^67,95^ Taiwan stood out for its innovative PPP which, through a government-led centralised supply chain, mitigated the impacts of unpredictable disruptions, built supply chain resilience, and ensured mask availability to the public.^109^ After the PPP had fulfilled its objectives, the government ended the centralised operations and the supply chain returned to normal.^109^

####  Gatekeeping and Resource Distribution

 Governments acted as gatekeepers in managing the procurement and distribution of supplies.^22,28,43,57,63,75,97^ Effective gatekeeping, as seen in Taiwan’s mask distribution system, ensured equitable access to essential goods.^53,65,71,109^ In other cases, lack of coordination led to delays and inequitable distribution.^7,28,49,58,88^

###  New Strategic Procurement

 The COVID-19 pandemic crisis revealed significant gaps in procurement practices, necessitating a shift toward strategic thinking, skill development, and adaptive methods for sourcing essential supplies. The following synthesis explores the key strategic procurement measures implemented, highlighting shifts in skills, workforce centralisation and supplier diversification.

####  Strategic Shift Requiring New Skills

 For procurement to become truly strategic, it necessitated elevating the role of the purchasing function and equipping procurement teams with specialized skills and resources. This shift enabled procurement professionals to better assess healthcare needs and market offerings.^23^ Integrating makers, engineers, and other technical experts into procurement teams enhanced the ability to evaluate non-traditional product designs effectively.^48^

####  Centralisation of Workforce and Designated Roles

 Centralised procurement professionals played a pivotal role in ensuring effective service provision during the crisis.^26^ However, in some cases, such as in emergency procurement scenarios, experts from centralised centres were excluded, limiting efficiency.^95^ The assignment of clear roles and qualified appointments, particularly in leadership positions, proved essential for managing supply chains strategically.^45^

####  Strategic Supplier Diversification and New Sourcing Approaches

 The urgency of the pandemic drove governments to adopt innovative approaches to sourcing supplies. Traditional suppliers were supplemented by new and alternative sources, often through expedited processes.^42^ For instance, the European Commission issued accelerated tenders for PPE and medical supplies, while procurement authorities engaged in direct outreach to potential suppliers within and outside the EU.^35,98,100^ These efforts included employing agents, leveraging digital tools, and coordinating with local businesses to secure supplies.^69,90,92,98,100^ Scotland^69^ and Newfoundland and Labrador^92^ were strong examples of strategic supplier diversification and new sourcing approaches through local collaborations. Scotland pursued redundancy through “buy and make” strategies, creating buffer stocks and promoting local manufacturing.^69^ This dual approach enhanced supply chain resilience but increased workloads due to the due diligence required.^69^ The US Joint Acquisition Task Force exemplified a rapid response in vendor-risk assessment and supplier diversification.^22^

####  Strategic Safeguards and Stockpiling

 To mitigate future risks, governments implemented strategic safeguards, such as guaranteed purchase agreements to support manufacturers and maintain national stockpiles.^37^ In Korea, for example, the government committed to purchasing surplus PPE production to stabilize supply chains.^37^ Similarly, the US and UK replenished their ventilator stockpiles through large government contracts.^82^ However, the US did not give the assurances to manufacturers that the Korean government did, and as large manufacturers ramped up ventilator production under the US DPA, Health and Human Services reported that ventilator stocks were sufficient and that it would be cancelling some production contracts.^88^ In the recovery phase, the Québec Ministry of Health’s Logistics Directorate established a national PPE reserve, negotiated a long-term contract for the supply of N-95 surgical masks from a local producer, and established a permanent supplier committee to help mitigate the potential effects of another PPE management crisis.^75^ Thus, such measures variably sought to balance production incentives with long-term inventory management to prepare for future public health emergencies.

###  Facilitating Domestic PPE Supply Chains

 The COVID-19 pandemic exposed vulnerabilities in global supply chains, prompting governments to adopt strategies to facilitate domestic production of PPE and medical supplies. These strategies encompassed retooling industries, increasing production capacity, providing financial incentives, simplifying regulations, and creating sustainable supply chains.

####  Retooling and Shifting Industrial Production

 To address immediate PPE shortages, governments encouraged and supported the retooling of existing industries to manufacture medical supplies. In countries like Canada, Ireland, the UK, and the US, distilleries were reconfigured to produce hand sanitizer, while clothing and garment manufacturers pivoted to making face masks.^47^ Automotive industries transitioned to producing ventilators,^91^ and factories in Thailand, the Philippines, and Vietnam retrofitted their facilities to manufacture PPE.^81^ These efforts required public funding and governments to assume significant risk to ensure smooth transitions and alleviate procurement delays.^23^

####  Increasing Domestic PPE Production

 Governments took proactive measures to ramp up domestic PPE production.^93^ In the US, partnerships with the private sector were sought to scale up production.^27^ Taiwan’s swift response involved deploying 60 new mask production lines, increasing daily mask output significantly.^53^ China rapidly expanded PPE production, supplying up to 83% of global PPE in May 2020.^80^ Advanced manufacturing technologies, such as 3D printing and robotics, were also employed to decentralise production and meet demand efficiently.^31^

 Strategic autonomy and reshoring became political priorities in many countries, including Spain and the EU, as they sought to reduce reliance on global supply chains by promoting local and regional manufacturing.^66,91^ Despite these efforts, policy missteps, such as the failure to fully utilize small manufacturing firms in the US, delayed the scaling of production during critical periods.^88^

####  Financial Incentives and Stimulus Measures

 To stimulate domestic production, governments deployed financial incentives and subsidies. In the US, the CARES Act allocated billions to support manufacturers, including $1.2 billion for PPE production and $10 billion for Operation Warp Speed.^72,77,80,88^ Taiwan and Korea provided funds to upgrade production lines and expedited licensing for companies that shifted production to masks.^37,71^ India offered financial incentives to micro, small, and medium enterprises to bolster self-reliance in manufacturing.^31^ These fiscal measures were crucial in encouraging companies to retool, expand capacity, and sustain production during the crisis.

####  Simplifying Regulatory Procedures

 Governments expedited market authorisation processes to accelerate the availability of PPE.^37,62,86,91,93^ The European Commission’s Recommendation 2020/403 allowed PPE to be marketed even if conformity assessments were incomplete.^62^ Countries like Spain authorised the sale of PPE without CE markings, while other nations such as Belgium, England, and Italy relaxed regulations to speed up production.^93^ Fast-tracking certifications and approvals enabled manufacturers to swiftly pivot and meet demand, ensuring critical supplies reached healthcare providers without unnecessary delays.^86^

####  Creating Sustainable Domestic Supply Chains

 In response to the crisis, efforts were made to establish long-term, sustainable domestic supply chains. The Biden administration issued executive orders to strengthen public health supply chains and reduce reliance on global markets.^77^ The goal was to create automated local mass production facilities, thereby enhancing supply chain resilience and lowering costs.^87^ The UK Government’s strategy of funding innovation and supply chain compression and regulatory facilitation was successful in creating local sourcing channels by substitution (this led to increased local availability of PPE, particularly ventilators).^96^ Other key examples of successful strategies in the literature were Korea’s reverse transcription polymerase chain reaction test kit strategy,^67^ the “make” component of Scotland’s “buy-and-make” strategy,^69^ Newfoundland and Labrador’s local manufacturing capacity strategies,^92^ and the UK’s Ventilator Challenge (utilisation of domestic resources from various industries for ventilator production).^96^

###  Local Solutions 

 The global supply chain vulnerability also prompted innovative local solutions to meet the urgent need for PPE and medical supplies. These localized approaches leveraged regional capacities, collaborative networks, and advanced technologies to mitigate supply disruptions.

####  Collaborative Local Networks and Task Forces

 In regions like Newfoundland and Labrador, healthcare supply chain teams collaborated with clinical leaders, Infection Prevention and Control teams, and Occupational Health and Safety specialists to inform PPE sourcing decisions.^92^ This coordinated approach involved leveraging local information technology infrastructure to create enhancements of supply chain visibility to improve decision-making.^92^ When traditional sourcing avenues were exhausted, task forces pivoted towards establishing local manufacturing capacities, resulting in the production of surgical masks, face shields, and medical gowns.^92^

####  Localized Production and Bespoke Solutions

 Local manufacturing and bespoke production played critical roles in addressing PPE shortages.^30,44,48,69,78,87^ In Ireland, smart communication channels improved supply chains, while bespoke PPE production filled specific gaps.^30^ Innovations included the use of sterilization and high-level disinfection techniques, such as vaporized hydrogen peroxide and ultraviolet irradiation, to safely reuse PPE.^30^ These contingency measures ensured continued availability of critical supplies even during peak shortages.

####  Additive Manufacturing and 3D Printing

 Advanced manufacturing and 3D printing emerged as crucial local solutions for addressing PPE shortages. In Italy, a regional hospital in Brescia successfully mitigated supply risks by 3D printing Venturi valves for ventilators.^87^ In Ireland, researchers and scientists crowdfunded initiatives to develop easy-to-build ventilators, while University College Dublin and IT Sligo produced ventilators using 3D printers and off-the-shelf components.^30^ Facial visors and other PPE components were also produced locally using 3D printing technologies for distribution to regional hospitals and nursing homes.^30^

 In the US and other countries, advanced manufacturing was employed to manufacture face shields, swabs, and mask brackets, providing rapid and adaptable solutions to supply chain disruptions.^48^ These bottom-up, grassroots innovations often outpaced centralised responses and demonstrated the value of community-driven problem-solving during crises.^66^

####  Local Adaptation and Redundancy Strategies

 Resilience in local supply chains was possible through multiple paths to supply resilience contingent on redundant capacity and local sourcing options at the start of the pandemic.^96^ In a multiple case study of the UK, Switzerland and Germany, Dube and colleagues found that low redundancy combined with limited local sourcing options was associated with more diverse strategies and flexibility, whereas high redundancy combined with multiple local sourcing options was associated with more focused strategies and agility.^96^ For instance, in Scotland, supply resilience was enhanced through redundancy strategies, combining “buy” and “make” approaches, where collaboration between government agencies and quasi-autonomous non-governmental organisations (quangos) enabled the establishment of entirely new supply chains for PPE.^69^ This strategy relied on the willingness of local companies to contribute to the national effort, reflecting a strong community-driven response.^69^

###  Information and Inventory Management 

 Robust information and inventory management systems played an important role in maintaining an effective supply chain for PPE and medical supplies. Successful strategies included systematic information coordination, digital tracking tools, optimized inventory networks, and dynamic demand prioritisation, however information constraints were limiting.

####  Systematic Information Management

 Effective information management proved essential in optimizing emergency logistics and supply chain coordination. In China, systematic information management facilitated coordination between production enterprises, logistics providers, and government agencies, ensuring the timely distribution of supplies and medical equipment through a dynamic demand-based system.^33^ Countries like Denmark, Norway, and England introduced national monitoring systems to manage the reporting, allocation, and distribution of PPE.^93^

####  Inventory Management and Digital Solutions

 Accurate inventory management was crucial for addressing PPE shortages and maintaining supply chain stability. Provinces in Canada, such as British Columbia, Alberta, and Newfoundland and Labrador,^92^ adopted digital inventory management tools to monitor PPE stockpiles and support decision-making.^49^ However, in regions like Ontario, reliance on manual counting and reporting of PPE supplies created uncertainty and eroded workforce confidence.^46,49^ Additionally, digital solutions such as web-based intensive care registers enabled hospitals to monitor PPE stockpiles and manage surge capacity effectively.^93^

####  Prioritisation and Allocation Strategies

 In Europe, during the early stages of the pandemic, hospitals were often prioritised over other healthcare settings, such as care homes, due to the immediate need for protective equipment in acute care environments.^49,79^ Germany stood out from other European countries by adopting a more integrated approach prioritising both hospitals and care homes for PPE allocation.^79^

####  Constraints of Information and Standards

 Initial expectations for procurement coordination were tempered by the reality of information constraints within fragmented healthcare systems.^63^ Ministers and officials faced challenges due to the lack of standardised data across diverse suppliers and healthcare providers.^63^ As shown through interviews with health system leaders in Canadian provinces, most reported inconsistent adoption of global product standards, which impeded their abilities to identify products, verify their attributes and trace them from manufacturer to patient care.^49^

###  Equity, Fair Dealing, and Public Trust 

 Achieving fair distribution of PPE and medical supplies while maintaining accountability and fostering public confidence in public procurement processes proved to be both critical and challenging.

####  Equitable Access and Fair Distribution

 Efforts to ensure equitable access to medical supplies were central to global and national responses. The 73rd World Health Assembly passed a resolution advocating for the fair distribution of drugs, medical supplies, and equipment to combat COVID-19.^76^ This global commitment underscored the importance of addressing disparities in access, especially as wealthier nations often secured supplies at the expense of poorer countries.^85^ For instance, the U.S. monopoly over remdesivir supplies in July 2020 highlighted the inequities in global procurement, adversely affecting supply availability for other regions.^50^

 Domestically, some regions implemented fair distribution mechanisms to achieve equity. For example, the name-based mask rationing system in Taiwan ensured universal access to masks by supplying them fairly across the population.^65^ However, competition between subnational entities, such as US states, drove up contract prices and exacerbated inequities, with wealthier states like New York and California often outbidding smaller states.^23,27,58^

####  Public Trust and Satisfaction in Government Responses

 The effectiveness of government responses to PPE procurement significantly influenced public trust and satisfaction. In Macao, the government’s transparent dissemination of information about mask supply schemes fostered public confidence, despite initial challenges.^89^ Measures such as maintaining social order during mask distribution and ensuring product quality were closely scrutinized by the public, reflecting the importance of transparency in maintaining trust.^89^

####  Transparency and Accountability in Procurement

 In contrast, failures in procurement transparency and fairness led to the erosion of public trust.^26^ In some cases, governments failed to adhere to established rules, leading to mismanagement and conflicts of interest.^41^ The misuse of public funds, lack of competitive bidding processes (designed to reduce fraud and ensure fair dealing), and perceptions of cronyism in PPE contracts undermined citizens’ confidence in public institutions.^35,41,42,68,84,98^ In Romania, direct procurement during the pandemic allowed for minimal transparency, giving the impression that accountability was forfeited and the failure to publish contract award details in accordance with legal requirements further undermined trust and transparency.^35^ The Autonomous Municipal Government of Pastaza in Ecuador bypassed control procedures, generating risk and compromising institutional interests.^55^

 Nevertheless, mechanisms to enhance transparency were implemented in some contexts. The US government, for instance, mandated public reporting of national equipment assessments and the transparent distribution of supplies under the DPA.^22^ Audit mechanisms, such as those performed by the Court of Auditors, were recommended to ensure that procurement decisions remained transparent and accountable^73^ and other entities, like the State Medical Corporation Ltd. (In Odisha province, India), ensured adherence to protocols with thorough documentation and audits to maintain accountability.^83^ A formal audit of Dutch PPE procurement, showed various accountability issues, such as a bank guarantee issued without formal approval.^98^

## Discussion

 Across a broadly global literature, covering healthcare procurement responses from all levels of government and public authority to the COVID-19 pandemic, this research has mapped these responses to the public procurement ecosystem, consisting of the governmental framework and context, public procurement system, and process and methods, as described by Thai^8^ and van Weele.^10^

 The scale and impact of the COVID-19 pandemic required governments to take action well outside of the public procurement processes and methods themselves, as defined by van Weele,^10^ to procure and distribute the required supplies. Governments had to change the structures of public procurement organisations, reorganize public procurement workforces, and adapt or adopt regulations within their systems.

 Moreover, they had to make significant changes to the contexts in which their public procurement systems operate. In most cases, this first meant a change to the legal context by invoking crisis or emergency legislation which then enabled swift responses in other contextual areas such as international trade policy, to control the flow of critical medical goods, and economic and market conditions with adaptive strategies for facilitating domestic PPE supply chains, retooling industries, increasing production through financial incentives, simplifying regulations, and creating sustainable domestic supply chains.

 At low levels of governance, local solutions played a pivotal role in mitigating PPE shortages and supply chain disruptions. Through collaborative networks, additive manufacturing, bespoke production, and technical innovations, communities demonstrated remarkable adaptability and resilience. Bottom-up initiatives not only addressed immediate needs but also highlighted the importance of localized capacity-building and innovation in enhancing supply chain robustness for future health emergencies.

 Notwithstanding the significant attention to contextual and system-level responses in the selected literature, there were also significant responses at the procurement process and methods level. Overall, the expedited procurement strategies employed during the pandemic varied widely across governments, balancing the need for speed and flexibility with the necessity for quality assurance and accountability. The rapid relaxation of procurement rules and procedures underscored the urgency of ensuring the availability of critical supplies yet also highlighted the risks of insufficient regulatory oversight during times of crisis. The strategic procurement response, on the other hand, involved a multifaceted approach, including skill enhancement, workforce centralisation, supplier diversification, and extraordinary measures to secure supplies. The crisis revealed the importance of adaptive strategies, proactive safeguards, and collaborative efforts to navigate supply chain disruptions.

 These approaches are consistent with the inherent trade-offs between policy objectives or outcomes, conformance to regulations and fair dealing, and efficiency and value for money that are central to the procurement management framework of Schapper and colleagues,^11^ and also with the observations of Dewick and colleagues from their study of procurement strategies of multinational corporations during the COVID-19 pandemic.^110^ Dewick and colleagues observed two different views of how pandemic-induced supply disruptions would influence procurement strategy.^110^ The predominant view was of a temporary spike that would eventually return to normal pre-pandemic operations, which gave rise to quick and temporary strategies. The alternate view was of lasting changes in decision-making and other processes related to procurement strategy development, which gave rise to longer-term strategies.^110^ This review has shown that both acute emergency and longer-term strategic responses were present in public healthcare procurement during COVID-19, and that more serious accountability trade-offs were made in expedited procurement rather than strategic procurement responses. Recommendations from the literature suggest that governments must refine emergency procurement frameworks to better balance speed, equity, and accountability, to ensure more resilient, adaptable, and responsive supply chains in future crises. This could include such safeguards as framework agreements to readily respond to and minimize the need for, or negative impacts of, such trade-offs.^12^

###  Contribution to Purchasing and Supply-Chain Management Theory and Procurement Practice

 This research contributes to the literature on procurement management under crisis conditions by proposing the Public Procurement Ecosystem from descriptions by Thai,^8^ and van Weele,^10^ and enriching it with the inductive identification of 110 strategic areas within the 21 fields of the ecosystem. Further, it has identified 10 themes which cut across the strategic areas and serve to summarise and synthesise the public procurement strategies used by governments and public purchasers for the procurement of PPE and healthcare supplies during the COVID-19 pandemic. It is expected that future case comparison studies could use this ecosystem to characterise and compare cases.

###  Strengths, Limitations, and Opportunities for Future Research

 Strengths of this research included the adoption of a comprehensive analytical framework, and the rigorous scoping review methodology which was the best approach for achieving the aim of this review. This approach, however, traded-off broad scope for limited depth. A richer dataset of strategy characteristics, and thus deeper insights on mechanisms within the procurement processes, would best be achieved through case study methodology using interviews and document analysis, compared to what was possible through article extraction from a systematic search.

## Conclusion

 This scoping review of public procurement strategies during the COVID-19 pandemic has demonstrated a focus within the literature not only on the public procurement processes and methods themselves, but on governmental actions to adapt both structures of public procurement systems and conditions within broader environmental contexts to facilitate procurement goals. This synthesis highlights that effective procurement during a global crisis requires a balance of centralised oversight, decentralised execution, strategic partnerships, and international cooperation. In the context of global product shortages or maldistribution, public procurement was forced to trade off accountability and in many cases value-for-money, for efficiency and outcomes. Taken together, the literature suggests opportunities for the development of pre-planned safeguard mechanisms, such as framework agreements, to minimize the need for, and negative impacts of, such trade-offs.

## Acknowledgements

 The authors acknowledge André Gerges for his contributions to article screening, selection, and data charting.

## Ethical issues

 This research was exempt from the requirement of ethics committee approval.

## Conflicts of interest

 Authors declare that they have no conflicts of interest. Fonds de recherche du Québec – Société et Culture had no role in the conduct of the research.

## Disclaimers

 An earlier version of this research was presented orally at the 6th International Conference on Public Policy (ICPP6) in Toronto, Canada on June 29, 2023, but not published in document form.

## Endnotes


^[1]^ One of the studies included in the scoping review (Bikkina et al^44^) was formally withdrawn after this article was accepted for publication. Its inclusion reflects both the state of the literature and the inclusion criteria at the time of the review. This does not affect the validity of the analysis, discussion, or conclusions presented.

## Supplementary files


Supplementary file 1. Full Search Strategy.

Supplementary file 2. Extraction Chart and Public Procurement Ecosystem Mapping.
