# Supplementary material for: Public Healthcare Procurement Strategies in Response to the COVID-19 Pandemic: A Scoping Review
Source: Int J Health Policy Manag. 2025 Aug 10;14:8556. doi: 10.34172/ijhpm.8556 (PMC12573135; doi:10.34172/ijhpm.8556)
Supplement: Supplementary file 1 — Full Search Strategy. [file ijhpm-14-8556-s001.pdf]

Article title: Public Healthcare Procurement Strategies in Response to the COVID-19 Pandemic: A Scoping Review

Journal name: International Journal of Health Policy and Management (IJHPM)

Authors' information: Pierre-André Hudon[1]*, Matthew T. Haren[1,2,3], Jean-Baptiste Gartner[1,2,3,4,5,6], Frédéric Bergeron[7], André Côté[1,2,3,4,5,6]

[1]Département de management, Faculté des sciences de l'administration, Université Laval, Québec City, QC, Canada.

[2]Centre de recherche en gestion des services de santé, Université Laval, Québec City, QC, Canada.

[3]Centre de recherche de l'Institut de Cardio-Pneumologie de Québec, Université Laval, Québec City, QC, Canada.

[4]Centre de recherche du CHU de Québec, Université Laval, Québec City, QC, Canada.

[5]Centre de recherche du CIUSSS de Chaudière-Appalaches, Québec City, QC, Canada.

[6]VITAM, Centre de recherche en santé durable, Université Laval, Québec City, QC, Canada.

[7]Bibliothèque-Direction des services-conseils, Université Laval, Québec City, QC, Canada.

***Correspondence to:** Pierre-André Hudon; Email: pierre-andre.hudon@fsa.ulaval.ca

Citation: Hudon PA, Haren MT, Gartner JB, Bergeron F, Côté A. Public healthcare procurement strategies in response to the COVID-19 pandemic: a scoping review. Int J Health Policy Manag. 2025;14:8556. doi:10.34172/ijhpm.8556

Supplementary file 1: Full search strategy

Table S1: Full search strategy

Table S2: PRESS checklist

Table S3: PRISMA-ScR checklist

Table S1: Full search strategy

Medline (Ovid)

Date of the first search: 04-10-2021

Date of the first updated search: 23-11-2022

Date of the last updated search:12-03-2025

Database limit: publications from 2019 to 12 March 2025, were included.

#	Search strategy	Results
1	(procurement adj3 (public OR government or state*)).ti,ab,kw	
2	((medical or medicines or medication OR "personal protect* equipment" OR PPE OR "Safety device*" OR "protective device*" OR Mask* OR N95) adj2 (logistic* OR suppl* OR purchas* OR procur*)).ti,ab,kw	
3	"Equipment and Supplies"/sd OR "Equipment and Supplies, Hospital"/sd OR Personal Protective Equipment/sd OR "Protective Devices"/sd OR Purchasing, Hospital/sd	
4	2 OR 3	
5	Coronavirus Infections/ OR COVID-19/	
6	(pandemic* OR "COVID-19" OR coronavirus).ti,ab,kw	
7	5 OR 6	
8	(govern* OR states OR public OR nation* OR hospital OR federal OR "healthcare facilit*").ti,ab,kw	
9	Public Health/	
10	8 OR 9	
11	1 AND 7	
12	4 AND 7 AND 10	
13	11 OR 12	
14	limit 13 to yr="2019-2021"	504
15	limit 13 to ed="20211005-20221123"	140
16	limit 13 to ez=20221124-20250312 OR limit 13 to dt=20221124-20250312 OR limit 13 to ed=20221124-20250312	230
**Total number of results**		874

Embase (Embase.com)

Date of the first search: 04-10-2021

Date of the first updated search: 23-11-2022

Date of the last updated search: 12-03-2025

Database limit: publications from 2019 to 12 March 2025, were included. Results have been limited to prepublications and Embase database only for the last updated search.

#	Search strategy	Results
1	(procurement NEAR/3 (public OR government or state*)):ti,ab,kw	
2	((medical or medicines or medication OR "personal protect* equipment" OR PPE OR "Safety device*" OR "protective device*" OR Mask* OR N95) NEAR/2 (logistic* OR suppl* OR purchas* OR procur*)):ti,ab,kw	
3	'hospital equipment'/exp OR 'medical resource shortage'/exp OR 'device shortage'/de OR 'protective equipment shortage'/de	
4	'purchasing'/exp OR 'supply and distribution'/de OR 'law'/de OR 'government regulation'/de OR 'legal aspect'/de	
5	#3 AND #4	
6	#5 OR #2	
7	'coronavirus disease 2019'/de OR 'pandemic'/de	
8	(pandemic* OR "COVID-19" OR coronavirus):ti,ab,kw	
9	#7 OR #8	
10	(govern* OR states OR Public OR nation* OR hospital OR federal OR "healthcare facilit*"):ti,ab,kw	
11	'government'/de OR "public health"/de	
12	#10 OR #11	
13	#1 AND #9	
14	#6 AND #9 AND #12	
15	#13 OR #14	
16	#15 AND [01-01-2019]/sd	357
17	#15 AND [05-10-2021]/sd	313
18	#15 AND [24-11-2022]/sd	
19	#16 AND [embase]/lim NOT ([embase]/lim AND [medline]/lim)	131
**Total number of results**		801

Web of Science

Date of the first search: 04-10-2021

Date of the first updated search: 23-11-2022

Date of the last updated search: 12-03-2025

Database limit: publications from 2019 to 12 March 2025, were included.

#	Search strategy	Results
1	TS=(procurement NEAR/3 (public OR government or state*))	
2	TS=((medical or medicines or medication OR "personal protect* equipment" OR PPE OR "Safety device*" OR "protective device*" OR Mask* OR N95) NEAR/2 (logistic* OR suppl* OR purchas* OR procur*))	
3	TS=(pandemic* OR "COVID-19" OR coronavirus)	
4	TS=(govern* OR states OR Public OR nation* OR hospital OR federal OR "healthcare facilit*")	
5	#1 AND #3	
6	#2 AND #3 AND #4	
7	#5 OR #6	
8	#7 AND PY=(2019-2021)	475
9	Publication database date limit	376
10	#7 AND LD=(2022-11-24/2025-03-12)	498
**Total number of results**		1 349

ABI/Inform (ProQuest)

Date of the first search: 04-10-2021

Date of the first updated search: 23-11-2022

Date of the last updated search: 12-03-2025

Database limit: publications from 2019 to 12 March 2025 were included & peer-reviewed journals limit has been applied

#	Search strategy	Results
1	TI,AB(procurement NEAR/3 (public OR government or state*))	
2	TI,AB((medical or medicines or medication OR "personal protect* equipment" OR PPE OR "Safety device*" OR "protective device*" OR Mask* OR N95) NEAR/2 (logistic* OR suppl* OR purchas* OR procur*))	
3	SU("Purchasing" OR "Purchasing contracts" OR "Equipment purchasing" OR "Supply chain management" OR "Supply chains" OR Logistics)	
4	SU("Personal protective equipment" OR "Medical equipment" OR "Medical supplies")	
5	[S3] AND [S4]	

6	[S2] OR [S5]	
7	SU("COVID-19")	
8	TI,AB(pandemic* OR "COVID-19" OR coronavirus)	
9	[S7] OR [S8]	
10	TI,AB(govern* OR states OR Public OR nation* OR hospital OR federal OR "healthcare facilit*")	
11	SU("Government" OR "State government" OR "Public health" OR "Government contracts")	
12	[S10] OR [S11]	
13	[S1] AND [S9]	
14	[S6] AND [S9] AND [S12]	
15	[S13] OR [S14]	
16	15 AND YR(2019-2021)	604
17	peer-reviewed journals database limit	98
18	15 AND YR(2021-2022)	578
19	peer-reviewed journals database limit	112
20	[S15] AND PD(20221123-20250312)	
21	peer-reviewed journals database limit	115
**Total number of results**		325

MedRxiv (https://www.medrxiv.org/)

Date of the first search: 22-03-2021

Date of the updated search: 23-11-2022

Database limit: MedRxiv database limit has been applied; search in title or abstract only

#	Search strategy	Results
1	"Public procurement" OR "government procurement" OR "state procurement"	2
2	government AND "Supply chains"	3
3	government AND Purchasing	11
4	Public AND "Supply chains"	16
5	Public AND Purchasing	37
	**Total number of results**	69

Table S2: PRESS checklist

PRESS 2015 Guideline Evidence-Based Checklist

X	Translation of the research question
X	Does the search strategy match the research question/PICO?
X	Are the search concepts clear?
X	Are there too many or too few PICO elements included?
X	Are the search concepts too narrow or too broad?
X	Does the search retrieve too many or too few records? (Please show number of hits per line.)
X	Are unconventional or complex strategies explained?
X	Boolean and proximity operators (these vary based on search service)
X	Are Boolean or proximity operators used correctly?
X	Is the use of nesting with brackets appropriate and effective for the search?
X	If NOT is used, is this likely to result in any unintended exclusions?
X	Could precision be improved by using proximity operators (eg, adjacent, near, within) or phrase searching instead of AND?
X	Is the width of proximity operators suitable (eg, might adj5 pick up more variants than adj2)?
X	Subject headings (database specific)
X	Are the subject headings relevant?
X	Are any relevant subject headings missing; for example, previous index terms?
X	Are any subject headings too broad or too narrow?
X	Are subject headings exploded where necessary and vice versa?
X	Are major headings (‘‘starring’’ or restrict to focus) used? If so, is there adequate justification?
X	Are subheadings missing?
X	Are subheadings attached to subject headings? (Floating subheadings may be preferred.)
X	Are floating subheadings relevant and used appropriately?
X	Are both subject headings and terms in free text (see the following) used for each concept?
X	Text word searching (free text)
X	Does the search include all spelling variants in free text (eg, UK vs. US spelling)?
X	Does the search include all synonyms or antonyms (eg, opposites)?
X	Does the search capture relevant truncation (ie, is truncation at the correct place)?
X	Is the truncation too broad or too narrow?

X	Are acronyms or abbreviations used appropriately? Do they capture irrelevant material? Are the full terms also included?
X	Are the keywords specific enough or too broad? Are too many or too few keywords used? Are stop words used?
X	Have the appropriate fields been searched; for example, is the choice of the text word fields (.tw.) or all fields (.af.) appropriate? Are there any other fields to be included or excluded (database specific)?
X	Should any long strings be broken into several shorter search statements?
X	Spelling, syntax, and line numbers
X	Are there any spelling errors?
X	Are there any errors in system syntax; for example, the use of a truncation symbol from a different search interface?
X	Are there incorrect line combinations or orphan lines (ie, lines that are not referred to in the final summation that could indicate an error in an AND or OR statement)?
X	Limits and filters
X	Are all limits and filters used appropriately and are they relevant given the research question?
X	Are all limits and filters used appropriately and are they relevant for the database?
X	Are any potentially helpful limits or filters missing? Are the limits or filters too broad or too narrow? Can any limits or filters be added or taken away?
X	Are sources cited for the filters used

Abbreviation: PICO, population/problem, intervention/exposure, comparison, outcome.

From reference [6]. © 2015 CADTH. Reprinted with permission.

Jessie McGowan, Margaret Sampson, Douglas M. Salzwedel, Elise Cogo, Vicki Foerster, Carol Lefebvre, PRESS Peer Review of Electronic Search Strategies: 2015 Guideline Statement, Journal of Clinical Epidemiology, Volume 75, 2016, Pages 40-46,

(6) J. McGowan, M. Sampson, D. Salzwedel, E. Cogo, V. Foerster, C. Lefebvre
PRESS–Peer Review of Electronic Search Strategies: 2015 Guideline Explanation & Elaboration (PRESS E&E)
CADTH, Ottawa (2016)
Ottawa

Table S3: PRISMA-ScR checklist

Preferred Reporting Items for Systematic reviews and Meta-Analyses extension for Scoping Reviews (PRISMA-ScR) Checklist

SECTION	ITEM	PRISMA-ScR CHECKLIST ITEM	REPORTED ON PAGE #
**TITLE**			
Title	1	Identify the report as a scoping review.	1
**ABSTRACT**			
Structured summary	2	Provide a structured summary that includes (as applicable): background, objectives, eligibility criteria, sources of evidence, charting methods, results, and conclusions that relate to the review questions and objectives.	1-2
**INTRODUCTION**			
Rationale	3	Describe the rationale for the review in the context of what is already known. Explain why the review questions/objectives lend themselves to a scoping review approach.	2-5
Objectives	4	Provide an explicit statement of the questions and objectives being addressed with reference to their key elements (e.g., population or participants, concepts, and context) or other relevant key elements used to conceptualize the review questions and/or objectives.	5
**METHODS**			
Protocol and registration	5	Indicate whether a review protocol exists; state if and where it can be accessed (e.g., a Web address); and if available, provide registration information, including the registration number.	5
Eligibility criteria	6	Specify characteristics of the sources of evidence used as eligibility criteria (e.g., years considered, language, and publication status), and provide a rationale.	5 & Table 1 (p.6)
Information sources*	7	Describe all information sources in the search (e.g., databases with dates of coverage and contact with authors to identify additional sources), as well as the date the most recent search was executed.	7

SECTION	ITEM	PRISMA-ScR CHECKLIST ITEM	REPORTED ON PAGE #
Search	8	Present the full electronic search strategy for at least 1 database, including any limits used, such that it could be repeated.	Supplementary file 1
Selection of sources of evidence†	9	State the process for selecting sources of evidence (i.e., screening and eligibility) included in the scoping review.	Table 1 (p.6) & 7
Data charting process‡	10	Describe the methods of charting data from the included sources of evidence (e.g., calibrated forms or forms that have been tested by the team before their use, and whether data charting was done independently or in duplicate) and any processes for obtaining and confirming data from investigators.	7-8
Data items	11	List and define all variables for which data were sought and any assumptions and simplifications made.	8
Critical appraisal of individual sources of evidence§	12	If done, provide a rationale for conducting a critical appraisal of included sources of evidence; describe the methods used and how this information was used in any data synthesis (if appropriate).	7
Synthesis of results	13	Describe the methods of handling and summarizing the data that were charted.	8
**RESULTS**			
Selection of sources of evidence	14	Give numbers of sources of evidence screened, assessed for eligibility, and included in the review, with reasons for exclusions at each stage, ideally using a flow diagram.	8-9 including Figure 2
Characteristics of sources of evidence	15	For each source of evidence, present characteristics for which data were charted and provide the citations.	9-10, Figure 3 (p.11), Supplementary file 2.
Critical appraisal within sources of evidence	16	If done, present data on critical appraisal of included sources of evidence (see item 12).	N/A
Results of individual sources of evidence	17	For each included source of evidence, present the relevant data that were charted that relate to the review questions and objectives.	Supplementary File 2

SECTION	ITEM	PRISMA-ScR CHECKLIST ITEM	REPORTED ON PAGE #
Synthesis of results	18	Summarize and/or present the charting results as they relate to the review questions and objectives.	11-29, Figure 4 (p.12), Table 2 (p.12-14)
**DISCUSSION**			
Summary of evidence	19	Summarize the main results (including an overview of concepts, themes, and types of evidence available), link to the review questions and objectives, and consider the relevance to key groups.	29-31
Limitations	20	Discuss the limitations of the scoping review process.	31
Conclusions	21	Provide a general interpretation of the results with respect to the review questions and objectives, as well as potential implications and/or next steps.	31-32
**FUNDING**			
Funding	22	Describe sources of funding for the included sources of evidence, as well as sources of funding for the scoping review. Describe the role of the funders of the scoping review.	Title page and disclosure form

JBI = Joanna Briggs Institute; PRISMA-ScR = Preferred Reporting Items for Systematic reviews and Meta-Analyses extension for Scoping Reviews.

* Where *sources of evidence* (see second footnote) are compiled from, such as bibliographic databases, social media platforms, and Web sites.

† A more inclusive/heterogeneous term used to account for the different types of evidence or data sources (e.g., quantitative and/or qualitative research, expert opinion, and policy documents) that may be eligible in a scoping review as opposed to only studies. This is not to be confused with *information sources* (see first footnote).

‡ The frameworks by Arksey and O'Malley (6) and Levac and colleagues (7) and the JBI guidance (4, 5) refer to the process of data extraction in a scoping review as data charting.

§ The process of systematically examining research evidence to assess its validity, results, and relevance before using it to inform a decision. This term is used for items 12 and 19 instead of "risk of bias" (which is more applicable to systematic reviews of interventions) to include and acknowledge the various sources of evidence that may be used in a scoping review (e.g., quantitative and/or qualitative research, expert opinion, and policy document).

From: Tricco AC, Lillie E, Zarin W, O'Brien KK, Colquhoun H, Levac D, et al. PRISMA Extension for Scoping Reviews (PRISMAScR): Checklist and Explanation. Ann Intern Med. 2018;169:467–473. doi: 10.7326/M18-0850.
